# A Case of Auto-brewery Syndrome Treated with Micafungin

**DOI:** 10.7759/cureus.5904

**Published:** 2019-10-14

**Authors:** Jessie Saverimuttu, Fahad Malik, Marutha Arulthasan, Prasanna Wickremesinghe

**Affiliations:** 1 Infectious Disease, Richmond University Medical Center, Staten Island, USA; 2 Internal Medicine, University of Alabama at Birmingham, Montgomery, USA; 3 Internal Medicine, Richmond University Medical Center, Staten Island, USA; 4 Gastroenterology, Richmond University Medical Center, Staten Island, USA

**Keywords:** gut microbiota in health and disease, gut microbiota, antibiotics, probiotics, fermenter, alcohol

## Abstract

Auto-brewery syndrome is caused by alcohol brewing inside the human body; it is a rare clinical condition where the patient becomes inebriated without exogenous alcohol use. Yeast is responsible, and treatment requires an appropriate antifungal agent. If undiagnosed, the patient's life becomes a misery. We present a case of a 45-year-old male who suffered from this condition for over three years with two arrests for driving under the influence prior to being diagnosed. The patient stated that he felt the episodes were related to his meal intakes; therefore, he would skip most meals of the day. The patient visited several centers where he was told there was not much they could offer him and he was left without a diagnosis. A carbohydrate challenge test in a monitored setting showed elevated blood alcohol levels. He was treated with antifungals and a low carbohydrate diet which resulted in the resolution of his symptoms. Hence the importance of awareness among physicians is necessary along with a high index of suspicion.

## Introduction

Auto-brewery syndrome, also known as gut fermentation syndrome, drunkenness disease, and endogenous ethanol fermentation, is a rare clinical condition where the patient becomes inebriated without exogenous alcohol use. Alcohol is produced endogenously by microorganisms living in the patient’s gut. In most cases, yeast has been implicated as the causative organism [1-2]. Most articles published on this syndrome are anecdotal. Here, we present a proven case of auto-brewery syndrome with stool cultures demonstrating *Saccharomyces cerevisiae* and gastric and jejunal samples yielding *Candida intermedia* along with *Klebsiella pneumoniae* and* Enterococcus faecalis*. The patient’s symptoms resolved only with the use of an echinocandin.

## Case presentation

A 45-year-old Italian-American male with a body mass index (BMI) of 35 and a past medical history of type 2 diabetes mellitus, hypertension, and hyperlipidemia presented to our medical center. In August 2015, he was treated with antibiotics and steroids after a nasal septal surgery. He had been abstaining from alcohol use for the last 20 years. Two months after the antibiotic treatment, in October 2015, he experienced recurrent seizures and he appeared inebriated. This was the first event of his symptoms (Day 0). This raised suspicion among the physicians that the patient was a “closet alcoholic” and began treating him for alcohol withdrawal. The patient and his wife insisted that he was not consuming alcohol at any time. During this period, his wife had also noticed that he had slurred speech, glassy eyes, and the smell of alcohol in his breath. His sentences were not making sense and he had episodes of poor coordination and frequent falls.

A year after his first symptoms, he had extensive dental surgery; during this time period, he was given amoxicillin/clavulanic acid. This exacerbated his symptoms and continued to mimic alcohol intoxication. He had been repeatedly pulled off the road by the police for driving erratically. Due to this, the patient had begun using a breathalyzer to test his breath alcohol level before driving. Despite this precaution, he was arrested twice for driving while intoxicated. During each event, his blood-alcohol level was two to three times higher than the legal limit for intoxication. The second offense a month later led to a hospital admission on the same day due to his blood alcohol content being above 0.41% on the breathalyzer. This was equivalent to 410 mg/dl on a blood test. The blood alcohol content continued to rise while in the hospital despite the absence of alcohol consumption during the hospital stay. The patient was treated for alcohol intoxication and discharged.

**Figure 1:**
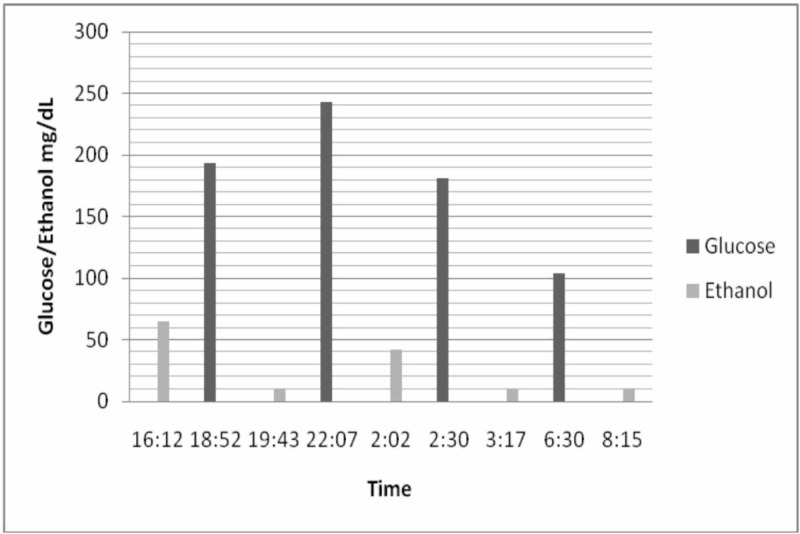
A graphical representation of blood ethanol levels in comparison to glucose

The patient and his wife attributed the symptoms to his meal intakes. The patient stated that he felt the episodes were related to his meal intakes; therefore, he would skip most meals of the day. The patient presented to a prior hospital in December 2016 before coming to our facility for confirmation of elevated blood alcohol levels in the setting of a secure hospital environment where alcohol beverages would not be obtainable (Table 1) (Figure 1). The hospital setting at that time provided the results as charted below. A full detailed history was obtained to determine any other possible causes. The patient denied using any unknown substances, chemicals, over the counter medications, any international medications, herbal medications or exogenous alcohol.

**Table 1:** Food intake with relationship to blood ethanol level in December 2016

Time	Activity	Ethanol Level
7:30 am	Wake up	0.20 mg%
12:00 pm	Ate 3 beef empanadas	
2:30 pm	Admitted to hospital	64.6 mg%
4:00 pm	Chicken noodle soup	
6:30 pm		<10 mg%
9:30 pm	High carb snacks	
10:00 pm	Still in Hospital	0.17 mg%
12:00 am		42.2 mg%
2:00 am	Fasting since 9:30pm	<10 mg%
6:00 am		<10 mg%
8:00 am	25 gram of carbs in yogurt + cake	
10:30 am		<10 mg%
2:00 pm	No food intake	
8:30 pm	Discharged	No follow up

The patient visited several centers where he was told there was not much they could offer him and he was left without a diagnosis. Due to the determination of the patient’s wife, he was finally able to find a physician who recommended using a carbohydrate challenge test in a monitored setting. This was a test in which a high load of carbohydrate or glucose is administered to the patient orally and then either breath alcohol or blood alcohol levels are tested in a monitored setting with timed intervals. This test was similar to glucose challenge testing for diabetes mellitus; higher glucose load of approximately 100 to 200 grams was used. The test showed that the patient’s ethanol levels had been fluctuating during his entire stay in the hospital.

The table below was obtained from our inpatient hospital monitored setting again in the absence of alcohol during two different hospital visits (Table 2). The test indicates that there was a presence of alcohol in the absence of exogenous alcohol. The urine was tested positive for alcohol metabolites like ethyl glucuronide and ethyl sulfate over 1,000 ng/mL. The patient underwent another complete carbohydrate challenge test with timed intervals at another institute to again monitor and confirm the blood alcohol levels over a 24-hour period to verify the diagnosis with even more certainty. The blood-alcohol level at that institute elevated to over 400 mg/dl at one hour and the study was halted. Stool studies that were obtained by deoxyribonucleic acid (DNA) by polymerase chain reaction (PCR) for stool profiling at that time revealed *Saccharomyces cerevisiae* (brewery yeast) with quintile distribution in the 3+ profile in addition to other bacterial organisms.

**Table 2:** Food intake with relationship to blood ethanol level in the absence of alcohol Ethanol levels elevated in the absence of exogenous alcohol with higher levels occurring temporally after glucose consumption; 200 grams of glucose was given 30 minutes prior to testing. Routine hospital food was provided during the testing with no carbohydrate restriction.

Time	Glucose (mg/dL)	Ethanol (mg/dL)
20:09	301	283
16:08	185	3
21:00	168	3
10:00	328	39
5:25	169	x
9:02	128	x
10:51	x	3
15:15	197	3
19:45	x	3
21:00	231	3
14:25	165	3

Time	Glucose (mg/dL)	Ethanol (mg/dL)
11:10	227	173
14:41	160	80
20:00	179	3
3:30	128	3

Finally, after about a year and two months since his first symptom, he was diagnosed with the auto-brewery syndrome. The patient was started on a high protein and a low carbohydrate diet. He was also placed on a three-week course of fluconazole at 100 mg orally daily along with a pure Lactobacillus probiotic. Subsequently, he was placed on multiple courses of fluconazole during the course of his treatment. Despite this, the patient continued to produce endogenous alcohol. A colonoscopy was performed by his gastroenterologist with a collection of stool samples which grew* Saccharomyces cerevisiae* from the small bowel within two weeks. He was started on another course of fluconazole, but the dose was increased to 200 mg orally daily. Even with the higher dose of antifungal therapy, he continued to be positive for alcohol by breathalyzer and blood testing. During this treatment, the patient developed severe vomiting and he was admitted to the intensive care unit for acute pancreatitis as a complication to endogenous alcohol production from his auto-brewery syndrome with an admission blood alcohol level of 173 mg/dl (0.173 mg% on the breathalyzer) and lipase level was 12,000 units. During the hospitalization, the patient underwent another upper endoscopy with gastric and jejunal samples growing *Candida intermedia, Klebsiella pneumoniae*, and *Enterococcus faecalis.*

Given the fact the patient was still positive for alcohol even while on the fluconazole and being critically ill, the decision was made to change oral fluconazole to intravenous micafungin in order to cover for possible azole-resistant C*andida*, although the minimal inhibitory concentration (MIC) for the *Candida intermedia* to fluconazole was 0.250 mg%. He was continued on the Lactobacillus containing probiotics. Once the micafungin treatment was started, each breathalyzer and blood alcohol content for the patient became undetectable. The intravenous micafungin was continued for eight weeks duration.

Our patient lost approximately 725 hours of work in sick time and disability time since 2015, but his story ends happily with him going back to work and leading a normal life, taking precaution in the use of antibiotics and avoid exposure to yeast in any form. Upper endoscopy was repeated after the completion of the micafungin; he no longer grew *Candida intermedia *but instead began to grow *Escherichia coli *and *Enterococcus faecalis*. Subsequently, the colonoscopy samples also did not grow any yeast species. A repeat upper endoscopy and lower colonoscopy samples a year later revealed a light growth of *Candida intermedia* but the patient was clinically asymptomatic. It was determined this was most likely recolonization of gut flora; therefore, he was just monitored with frequent physician visits, carbohydrate-free diet, probiotics, and home breathalyzer as needed.

## Discussion

Auto-brewery syndrome is a rare clinical entity. There is a dearth of medical literature about this entity. Some of the earliest cases of this syndrome were described in Japan in the 1970s [3]. A high index of suspicion is necessary to be able to diagnose it, and this calls for knowledge of this condition coupled with obtaining a detailed history and thorough clinical examination. Auto-brewery syndrome should be considered in the differential diagnosis in any patient who denies alcohol use but has a recurrence of alcohol intoxication after all other common possible causes have been excluded. It’s a possibility that the high carbohydrate meal intake would cause the symptoms for alcohol intoxication and complete fasting could have caused the alcohol withdrawal symptoms.

The causation of this syndrome could be multifactorial, but the microbial agent identified has been yeast, commonly *Saccharomyces cerevisiae* [4-5] also known as Brewer’s yeast. The use of antibiotics could result in yeast overgrowth in the intestines. Our patient used antibiotics prior to the onset of his symptoms; however, exposure to yeast in food or work environment could also be implicated [5-6]. It has also been postulated that reduced activity of enzymes responsible for ethanol metabolism in the liver which may be genetically linked could be responsible for this syndrome [2]. Endogenous alcohol production has also been shown to increase in patients with diabetes mellitus and cirrhosis [7]. Our patient has type 2 diabetes mellitus.

The diagnosis should be confirmed carbohydrate challenge test in a monitored setting. There are different protocols that have been formulated [4,8], but a universal protocol is yet to be established. There is no test that can distinguish endogenous from exogenous alcohol [2]; hence, the need for close observation during the carbohydrate challenge testing is necessary to establish the diagnosis. This test primarily requires a baseline ethanol level. Then a high load ingestion of carbohydrates or glucose is given orally and then with timed intervals (2, 4, 8, 16, and 24 hours), the presence of ethanol is measured either by breathalyzer or blood draws. The presence of any amount of ethanol with a confirmed absence of exogenous consumption of alcohol leads to this diagnosis. Once diagnosed, the patients should be placed on a high protein, low carbohydrate diet as well as a bacterial probiotic. Ideally gastric, jejunal, and stool samples should be obtained for fungal cultures prior to starting treatment.

Antifungal medications seem to be the mainstay of treatment for this syndrome. Our patient did have *Saccharomyces cerevisiae* in the stool sample but not in the gastric or jejunal sample. *Candida intermedia *continued to be present in the gastric and jejunal samples, despite treatment with fluconazole. This raises the possibility that there could have been a mixture of azole-sensitive and -resistant *Candida *in the gastric and jejunal samples which resulted in the failure to respond to fluconazole but eventually responded to the echinocandin micafungin. Alcohol is absorbed in the stomach and the small intestine. Therefore the presence of the *Candida intermedia* in the upper intestine in our patient makes it the likely causative agent along with *Saccharomyces cerevisiae* in the stool. The presence of common bacterial gastrointestinal flora *Klebsiella pneumonia* and *Enterococcus faecalis* after treatment with micafungin gives strength to the argument that the yeast was responsible for the fermentation and production of alcohol.

Ethanol can be produced by fermentation of pyruvate under anaerobic conditions as one of the biochemical pathways. This has been well studied in *Saccharomyces cerevisiae *and *Escherichia coli*. *Candida intermedia* is also known to have fermentation properties for alcohol but uncertain to what extent [9]. According to our hypothesis, it is possible that many of these yeasts are likely different strains with different catalysts changing in the host environments to produce ethanol [9-10]. Many of these fungi can be asymptomatic colonization in people to improve immunity in the gut and influence bacterial microbiota [11]. It is probably fungi overgrowth with the use of antibiotics which resulted in eradicating the bacterial flora to maintain normal homeostasis in the gut. It is also a possibility that this patient had expressed a mutated strain that needs to be further studied for further evaluation that flourished with the low gut bacterial microbiome [6].

## Conclusions

In conclusion, auto-brewery syndrome is a rare clinical condition where the patient becomes inebriated without exogenous alcohol use and is difficult to diagnose unless in a completely monitored setting. In our patient, every possible diagnosis was excluded prior to diagnosing him with auto-brewery syndrome. A thorough detailed history, along with all possible variables in diet and herbal supplements, were excluded. If this condition is left undiagnosed, the patient's life can become miserable. These patients can become alcohol dependent from the endogenous ethanol production and might not even recognize the symptoms. Endogenous ethanol production can have the effect on our body as exogenous alcohol. Hence, the importance of awareness among physicians, and a high index of suspicion are crucial in order to provide the appropriate care for these patients.
